# Cerebral air embolism associated with penetrating lung injury: a case report and review of the literature

**DOI:** 10.1002/ams2.250

**Published:** 2016-11-10

**Authors:** Ayumu Yamaoka, Kei Miyata, Eichi Narimatsu, Eiji Sakawaki, Sonoko Sakawaki, Suguru Hirayama, Shuji Uemura, Naoya Yama

**Affiliations:** ^1^ Department of Emergency Medicine Sapporo Medical University Sapporo Hokkaido Japan; ^2^ Department of Diagnostic Radiology Sapporo Medical University Sapporo Hokkaido Japan; ^3^ Present address: Emergency Medical Center Hakodate Municipal Hospital 1‐10‐1 Minato‐cho Hakodate 041‐8680 Japan

**Keywords:** Cerebral air embolism, penetrating lung injury, positive pressure ventilation, pulmonary vein bronchial fistula, trauma

## Abstract

**Case:**

A 44‐year‐old man intentionally stabbed himself in the anterior neck and left thorax with a fruit knife. Physical examination revealed two open wounds entering the thoracic cavity in the front chest, and a stab wound entering the trachea at the neck. Two chest tubes were initially inserted for the left lung injury with open hemopneumothorax. Nevertheless, the worsening oxygenation required positive pressure ventilation (PPV) with endotracheal intubation.

**Outcome:**

Right hemiparesis was found during weaning from PPV. Magnetic resonance imaging revealed multiple infarctions in the area of the bifrontal and right temporal lobes. Cerebral air embolism (CAE) was strongly suspected from the imaging findings and clinical course.

**Conclusion:**

We concluded that mechanical ventilation was strongly involved in the occurrence of CAE. If delayed abnormal neurological findings are observed in patients with penetrating lung injuries receiving PPV management, CAE should be considered.

## Introduction

Cerebral air embolism (CAE) is caused by air bubbles in the vascular system. These bubbles obstruct the intracranial blood vessels, and lead to symptoms of cerebral ischemia. Cerebral air embolism is commonly caused by arterial catheterization, barotrauma, decompression sickness, iatrogenic interventions, and trauma.^1^ Little is known about CAE associated with severe penetrating chest trauma. We report a rare case of delayed CAE caused by a chest stab wound, and review published works.

## Case

**Figure 1:**
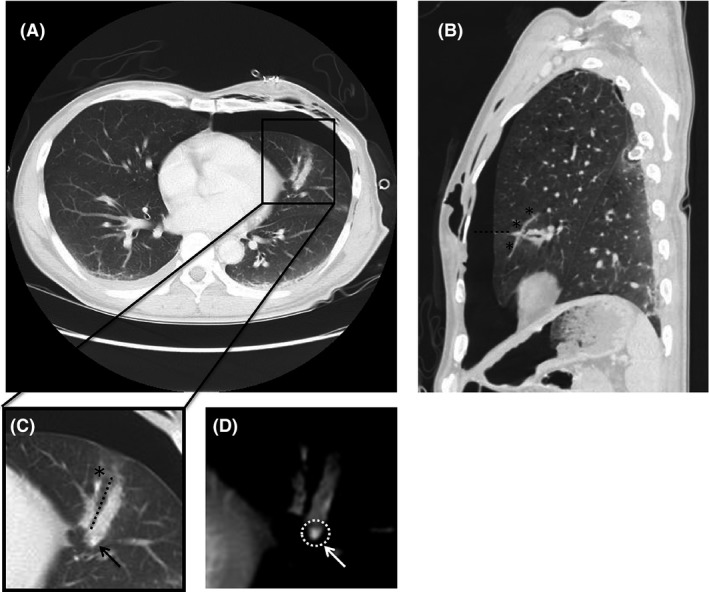
Axial (A) and sagittal (B) contrast‐enhanced computed tomography images of a 44‐year‐old man with self‐inflicted stab wounds to the anterior neck and left thorax. The images show the lung injury at the left lung lingular division (black square), and a pulmonary vein (black asterisk) around the lung injury (black dotted line). Magnified contrast‐enhanced computed tomography images of the arterial phase (C, D) show a pulmonary vein (black arrow, white arrow) and a pulmonary artery (black asterisk) near the consolidation of the lung injury (black dotted line).

**Figure 2:**
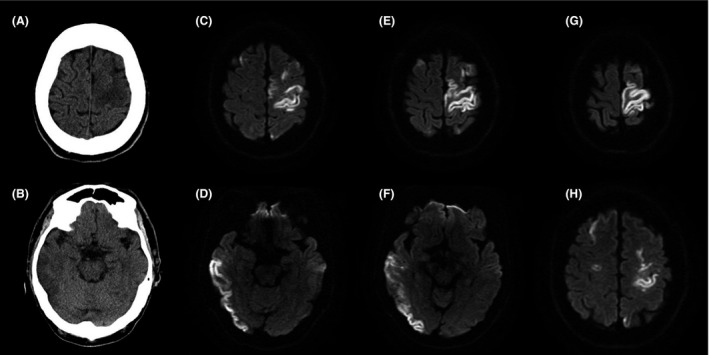
Computed tomography images of a 44‐year‐old man with self‐inflicted stab wounds to the anterior neck and left thorax. Images of the head (A, B) show cerebral infracts in the bifrontal lobe and the right temporal lobe. Magnetic resonance diffusion weighted images (C–H) show multiple high‐intensity lesions along the cortical layer.

A 44‐year‐old man with a history of schizophrenia intentionally stabbed himself in the anterior neck and left thorax with a fruit knife. On arrival to our hospital, he was alert and oriented without neurological abnormalities. His vital signs were respiratory rate 14 breaths/min, blood pressure 149/89 mmHg, and heart rate 90 b.p.m. His SpO_2_ was 97% using 10 L/min O_2_. Physical examination revealed three stab wounds: one was a neck wound (approximately 4 cm) that penetrated the trachea, and the others were chest wounds (approximately 1.5 cm and 4 cm) that entered the thoracic cavity from the left front chest. Chest radiograph revealed s.c. emphysema. Ultrasonography revealed pleural effusion in the left chest cavity. A left‐side chest tube was inserted, and the stab wounds in the left chest were sutured. Contrast‐enhanced computed tomography (CT) of the patient's neck and chest showed s.c. emphysema, pneumomediastinum, tracheal injury, right pneumothorax, left hemopneumothorax, and intrapulmonary hemorrhage. The abbreviated severity score of the chest was 3 points. The pulmonary vein and artery ran across the lung consolidation in the vicinity of the penetration route (Fig. 1). There were no abnormal findings such as vascular injury or intravascular air shadow on the 3D‐CT angiography of the head and neck from the aortic arch. Three hours later ICU admission, another chest tube was inserted in the left thoracic cavity due to progressive aggravation of s.c. emphysema and oxygenation. Despite the chest tubes, pneumothorax and oxygenation gradually worsened (SpO_2_ 78%, using 15 L/min O_2_). Six hours after hospital admission, endotracheal intubation was carried out to provide positive pressure ventilation (PPV) under sedation and analgesia. On the third day after admission, improved oxygenation allowed for extubation in the absence of persistent air leak from the chest cavity. However, right hemiparesis appeared following weaning from sedation and mechanical ventilation. The manual muscle test grade of the right upper and lower extremities was 1/5. Head CT showed subacute cerebral infarcts in multiple areas of the bifrontal and right temporal lobes. Diffusion weighted imaging (DWI) of the head showed right temporo‐occipital and left frontal cortical laminar hyperintensity (Fig. 2). Three‐dimensional CT angiography of the head and neck showed no evidence of stenosis or occlusion in major cerebral arteries. We did not detect paroxysmal atrial fibrillation on the electrocardiogram monitor. Laboratory analysis did not reveal systemic coagulation disorder or congenital coagulation defects. The presence of foramen ovale could not be determined. Taking into account the imaging findings and the clinical course of the patient, we diagnosed CAE from the penetrating lung injury that had occurred during PPV. Thirty milligrams of edaravone was given twice daily for 14 days. On the 18th day after admission, right hemiparesis improved after rehabilitation; the manual muscle test grade of the right upper extremity was 5/5 and that of the right lower extremity was 4/5. The patient was transferred to another hospital on the 56th day after admission.

## Discussion

The clinical course of our case suggested two important clinical issues. First, penetrating chest trauma could be a rare cause of late‐onset CAE. Second, PPV for patients with severe lung injury could be a trigger of CAE.

Among 14 reports retrieved by searching the electronic database PubMed with the key words ([“cerebral”(All Fields) OR “systemic”(All Fields)] AND “air embolism”(All Fields)]) AND (“trauma”[All Fields] OR “injury”[All Fields]), seven cases were diagnosed with CAE associated with blunt chest trauma on CT scans. The remaining seven cases received chest compressions for resuscitation (Table 1).^2^, ^3^, ^4^, ^5^, ^6^, ^7^, ^8^ Penetrating chest trauma could be a rare cause of late‐onset CAE. As the reason, we speculate that blunt chest trauma is pathologically severe and includes lung contusion whereas penetrating trauma often remains as a localized injury.

**Table 1:** Summary of published reports of patients with cerebral air embolism after chest trauma

Year	Primary author	Age, years/sex	Cause of injury	Type of injury	HX	PX	MRF	LC/PT	PPV	Detection of CA	Diagnosis of CI	Outcome
2002	Brownlow	37/M	MA	Blunt	−	+	+	+	+	−	Day 3	Full recovery
2012	Kesieme	46/F	Fall	Blunt	+	−	+	+	‐	−	Day 4	Full recovery
2014	Present case	44/M	Stabbing	Penetrate	+	+	−	+	+	−	Day 4	Minor disability
2016	Reith	28/M	MA	Blunt	−	−	−	+	+	+	Admission	Full recovery
1994	Muras	15/F	Fall	Blunt	+	+	+	+	−	+	Admission	Dead
2001	Sakai	75/M	MA	Blunt	−	−	−	+	−	+	Admission	Dead
2008	Milla	19/M	MA	Blunt	−	−	−	+	+	+	Admission	Dead
2011	Brederlau	13/M	TA	Blunt	−	−	+	+	+	+	Admission	Dead

CA, cerebral air; CI, cerebral infarction; F, female; HX, hemothorax; LC, lung contusion; M, male; MA, motorcycle accident; MRF, multiple rib fractures; PPV, positive pressure ventilation; PT, pneumatocele; PX, pneumothorax; TA, traffic accident.

The definitive diagnosis of CAE might be difficult to confirm in the acute phase, because the air shadow in the vessels of the brain in the CT examination can disappear in 0.5–30 h.^9^ Especially in trauma, the air shadow often disappears during the initial resuscitation treatment, imaging tests, surgery, and ventilator management.^4^, ^7^ Therefore, CAE could be suspected as a diagnosis of exclusion in the absence of intravascular air shadows. Instead, it has been reported that magnetic resonance imaging of CAEs specifically showed multiple cortical laminar necrosis.^10^ Although we could not detect cerebral air bubbles on the head CT, DWI findings indicated characteristic laminar cortical hyperintense lesions in multiple lobes. These radiological findings were consistent with a diagnosis of CAE. This suggests the possibility of the diagnosis of CAE in DWI of the subacute phase, if intravascular air shadows are not detected.

Positive pressure ventilation in a patient with severe lung injury could be a trigger of CAE. In trauma, the tearing of lung parenchyma might induce direct communication between the pulmonary vein and the bronchi.^1^ Systemic air embolism occurs due to the influx of air into the pulmonary venous system as a result of a positive gradient caused by low pulmonary venous pressure or increased airway pressure.^1^ Positive pressure ventilation can also lead to reversal of the pressure gradient of the pulmonary venous and airway pressure.^1^ Our case showed no neurological abnormalities from the time of admission to the introduction of PPV, and CT examination immediately after endotracheal intubation revealed no air shadows of the vessels. As such, we believe that ongoing PPV was strongly involved in the delayed onset of CAE. In our review of eight cases, PPV was undertaken in five patients with severe lung injuries, such as hemothorax, pneumothorax, multiple rib fractures, lung contusion, or pneumatocele. Therefore, we suggest that PPV for severe lung injury could be a risk factor in the delayed onset of cerebral infarction.

Additionally, routine follow‐up head CT scans are rarely carried out in victims with life‐threatening injuries or no neurological symptoms. It is speculated that the incidence of CAE in the critical case setting might be underestimated, which can delay definitive diagnosis. The late onset of CAE should be considered for patients with severe penetrating chest injuries.

## Conclusion

We experienced a rare case of CAE associated with penetrating lung injury. The clinical course suggested that the PPV in the intensive care unit was strongly involved in the occurrence of CAE. A definitive diagnosis of CAE is often delayed. Clinicians should note that mechanical ventilation can potentially result in delayed‐onset CAE in penetrating lung injury.

## Conflict of Interest

None Declared.
